# Clear Cell Papulosis Associated With Multiple Developmental Abnormalities of the Skin

**DOI:** 10.7759/cureus.95110

**Published:** 2025-10-21

**Authors:** Kazuhiro Kawai, Tomoko Fukushige, Naoko Baba, Hiroshi Uchimiya, Takuro Kanekura

**Affiliations:** 1 Department of Dermatology, Kagoshima University Graduate School of Medical and Dental Sciences, Kagoshima, JPN; 2 Department of Dermatology, Kido Hospital, Niigata, JPN; 3 Department of Dermatology, Uchimiya Clinic, Kagoshima, JPN

**Keywords:** clear cell papulosis, congenital anomaly, developmental abnormality, hair disorder, pigmentary disorder, skin, syndrome

## Abstract

Clear cell papulosis of the skin is a rare condition in children characterized by multiple small hypopigmented macules or flat papules, predominantly distributed in the pubic area and over the abdomen, chest, and axillae, along the milk lines, and by mucin- and cytokeratin 7-positive large clear cells within the epidermis. Associations of clear cell papulosis with other diseases or disorders have been rarely described. We present a case of clear cell papulosis associated with a congenital sacral dermoid cyst, nevus of Ota, presumed accessory breast tissue, thin and lightly pigmented hair, congenital triangular alopecia, and a posterior helical ear pit.

## Introduction

Clear cell papulosis (CCP) of the skin is a rare condition in children, clinically characterized by multiple small hypopigmented macules or flat papules, predominantly distributed in the pubic area and over the abdomen, chest, and axillae, along the milk lines [1]. Histopathologically, it is defined by mucin- and cytokeratin (CK) 7-positive large clear cells within the epidermis [1-3]. In most reported cases, the patients were of Asian descent, and based on reports of affected siblings [1,2,4], a possible genetic predisposition has been postulated. Associations of CCP with other diseases or disorders have been rarely described [1,2]. Here, we present a case of CCP associated with multiple developmental abnormalities of the skin.

## Case presentation

**Figure 1:**
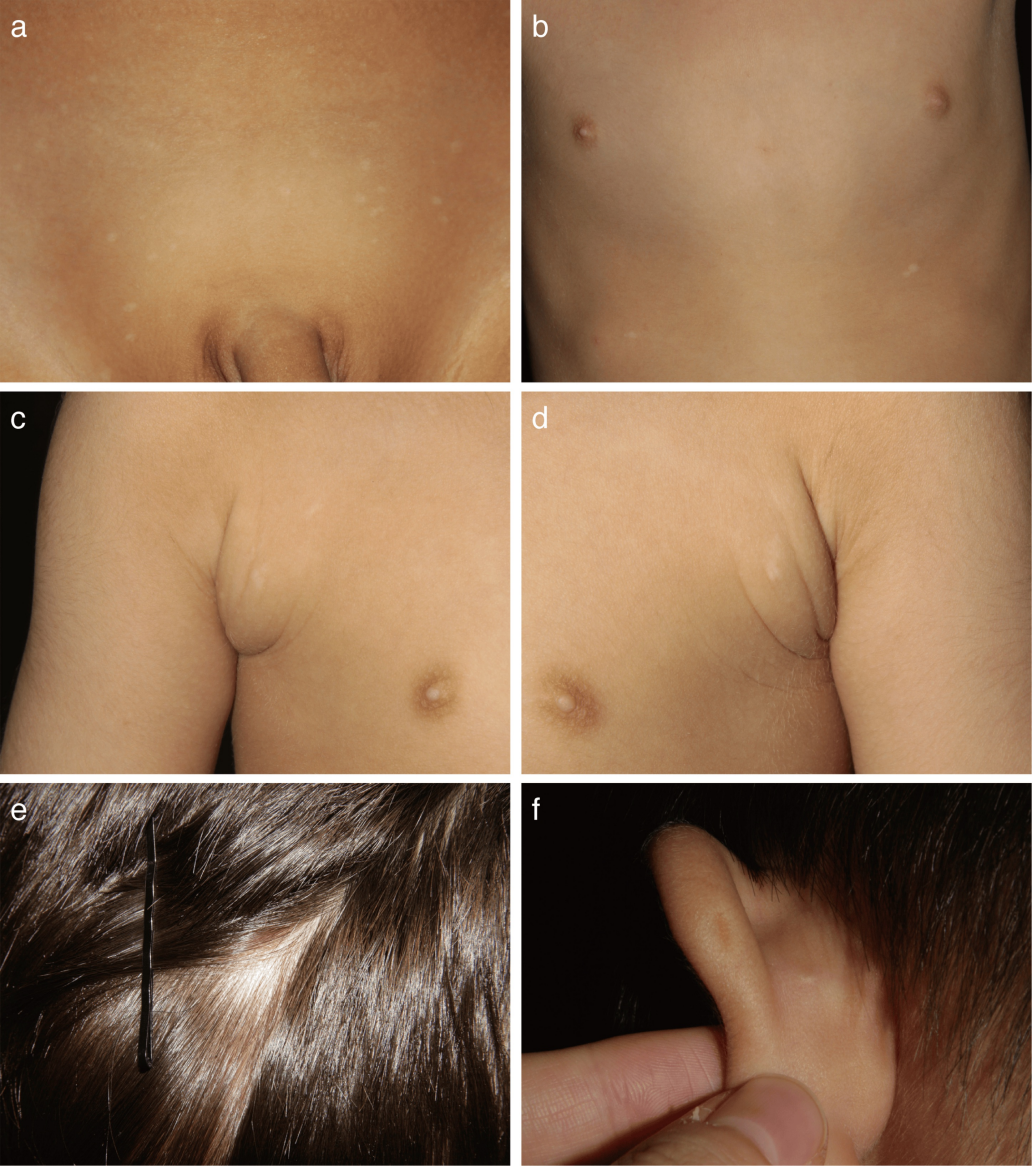
Clinical findings. (a) Multiple hypopigmented macules in the pubic area. (b) Hypopigmented macules on the abdomen and chest following the milk lines. (c,d) Subcutaneous soft masses with centrally located hypopigmented macules in the bilateral axilla. (e) An area of alopecia in the right frontotemporal region. (f) A pit in the posterior helix of the left ear.

A three-year-old Japanese boy presented with multiple small hypopigmented macules in the pubic area (Figure 1a). The lesions first appeared at the age of two and gradually increased in number. He was the second child of healthy, non-consanguineous Japanese parents. There was no family history of similar skin lesions. The mother reported no history of infection or medication use during pregnancy.

He had a congenital sacral dermoid cyst, which was surgically resected at nine months of age. He also had a nevus of Ota around the right eye since birth, which was treated with an alexandrite Q-switched laser.

Physical examination revealed hypopigmented macules scattered over the abdomen, chest, and bilateral axillae, distributed along the milk lines (Figures 1a-1d). Subcutaneous soft masses with centrally located hypopigmented macules, which were suggestive of accessory breast tissue, were present in bilateral axillae (Figures 1c, 1d), but were not biopsied. His scalp hair was thinner and less pigmented compared with that of his elder brother. An area of alopecia with vellus hairs was noted in the right frontotemporal region and diagnosed as congenital triangular alopecia (Figure 1e). A posterior helical pit was present in the left ear (Figure 1f).

**Figure 2:**
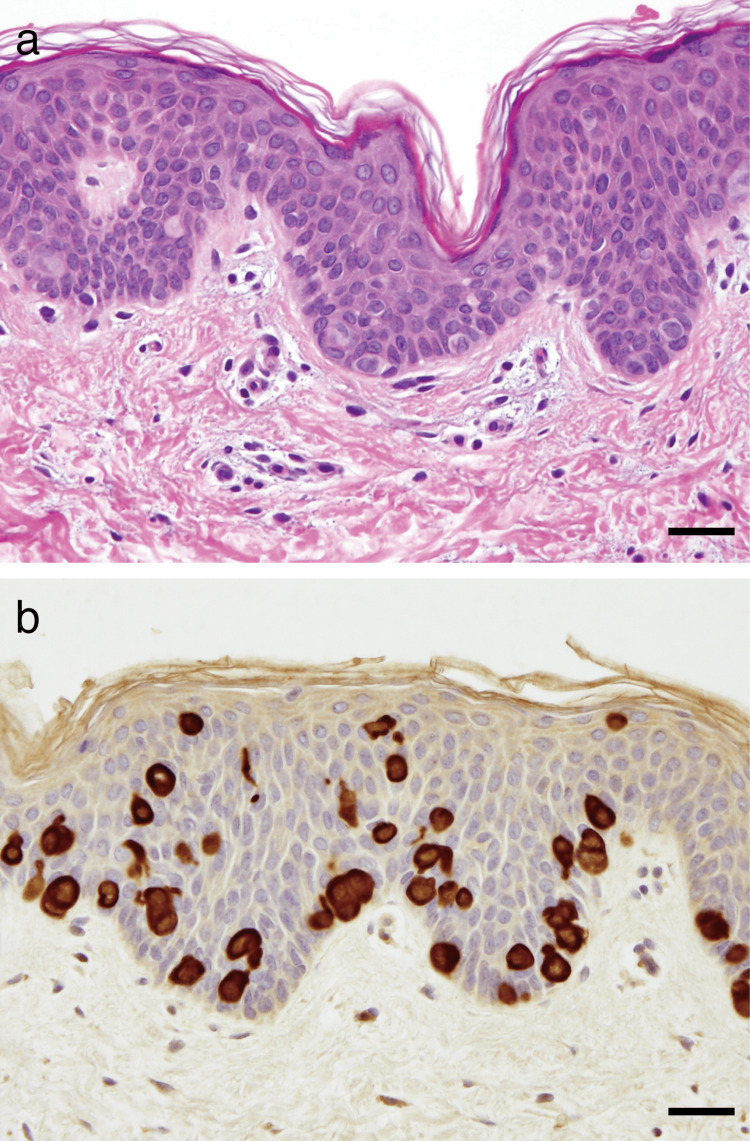
Histopathological and immunohistochemical findings. (a) Scattered clear cells in the epidermis (hematoxylin-eosin stain, scale bar = 30 μm). (b) The clear cells showed positive staining for cytokeratin (CK) 7 (scale bar = 30 μm).

A skin biopsy specimen from a depigmented macule revealed scattered large, clear cells within the epidermis (Figure 2a). They were positive for mucin stains, CK7 (Figure 2b), carcinoembryonic antigen (CEA), epithelial membrane antigen (EMA), and gross cystic disease fluid protein-15 (GCDFP-15), but were negative for CD1a and S100.

Based on these findings, a diagnosis of CCP was made. At the age of seven, the patient exhibited normal mental and physical development, and partial regression of the CCP lesions was observed.

## Discussion

A possible link between CCP and intraepithelial CK7-positive clear cells of Toker [5,6] has been proposed [1-4]. Although Toker cells were initially identified in the nipple epidermis [5], they are now considered a normal constituent of the epidermis along the milk lines [6]. Mucin stains are positive in CCP but negative in Toker cells [1-5], and most patients with CCP experience spontaneous regression [4]. Therefore, CCP may represent an abortive proliferation of aberrantly differentiated Toker cells. However, the precise pathogenesis of CCP remains unclear.

Our case is unique in that CCP was associated with multiple developmental abnormalities of the skin, including pigmentary and hair disorders. Although these associations might be coincidental, they share a common pathogenesis of impaired cell migration and/or apoptosis during development [7-9]. To our knowledge, similar syndromic associations with CCP have not been described, although a few cases of CCP associated with isolated pigmentary and/or hair disorders have been reported [1,2].

## Conclusions

We presented a case of CCP associated with a congenital sacral dermoid cyst, nevus of Ota, presumed accessory breast tissue, thin and lightly pigmented hair, congenital triangular alopecia, and a posterior helical ear pit. Since these associations are thought to arise from developmental abnormalities, a causal relationship between CCP and these disorders may exist. Accumulation of similar cases will be required to elucidate the detailed pathogenesis of CCP.

## References

[1] Kuo TT, Chan HL, Hsueh S (1987). Clear cell papulosis of the skin. A new entity with histogenetic implications for cutaneous Paget's disease. Am J Surg Pathol.

[2] Lee JY, Chao SC (1998). Clear cell papulosis of the skin. Br J Dermatol.

[3] Kumarasinghe SP, Chin GY, Kumarasinghe MP (2004). Clear cell papulosis of the skin: a case report from Singapore. Arch Pathol Lab Med.

[4] Tseng FW, Kuo TT, Lu PH, Chan HL, Chan MJ, Hui RC (2010). Long-term follow-up study of clear cell papulosis. J Am Acad Dermatol.

[5] Toker C (1970). Clear cells of the nipple epidermis. Cancer.

[6] Willman JH, Golitz LE, Fitzpatrick JE (2005). Vulvar clear cells of Toker: precursors of extramammary Paget's disease. Am J Dermatopathol.

[7] Wobser M, Hamm H (2019). Developmental anomalies. Harper’s Textbook of Pediatric Dermatology.

[8] Chan HH, Kono T (2003). Nevus of Ota: clinical aspects and management. Skinmed.

[9] Yin Li VC, Yesudian PD (2015). Congenital triangular alopecia. Int J Trichology.

